# Dynamic classification using credible intervals in longitudinal discriminant analysis

**DOI:** 10.1002/sim.7397

**Published:** 2017-08-01

**Authors:** David M. Hughes, Arnošt Komárek, Laura J. Bonnett, Gabriela Czanner, Marta García‐Fiñana

**Affiliations:** ^1^ Department of Biostatistics University of Liverpool Liverpool U.K.; ^2^ Department of Probability and Mathematical Statistics, Faculty of Mathematics and Physics Charles University Prague Czech Republic; ^3^ Department of Eye and Vision Science University of Liverpool Liverpool U.K.

**Keywords:** allocation scheme, credible intervals, longitudinal discriminant analysis

## Abstract

Recently developed methods of longitudinal discriminant analysis allow for classification of subjects into prespecified prognostic groups using longitudinal history of both continuous and discrete biomarkers. The classification uses Bayesian estimates of the group membership probabilities for each prognostic group. These estimates are derived from a multivariate generalised linear mixed model of the biomarker's longitudinal evolution in each of the groups and can be updated each time new data is available for a patient, providing a dynamic (over time) allocation scheme. However, the precision of the estimated group probabilities differs for each patient and also over time. This precision can be assessed by looking at credible intervals for the group membership probabilities. In this paper, we propose a new allocation rule that incorporates credible intervals for use in context of a dynamic longitudinal discriminant analysis and show that this can decrease the number of false positives in a prognostic test, improving the positive predictive value. We also establish that by leaving some patients unclassified for a certain period, the classification accuracy of those patients who are classified can be improved, giving increased confidence to clinicians in their decision making. Finally, we show that determining a stopping rule dynamically can be more accurate than specifying a set time point at which to decide on a patient's status. We illustrate our methodology using data from patients with epilepsy and show how patients who fail to achieve adequate seizure control are more accurately identified using credible intervals compared to existing methods.

## 1 INTRODUCTION

In many medical studies, measurements from patients are regularly taken over time on multiple clinical markers. Interest may be in the future prognosis of individual patients, eg, whether a patient will suffer renal graft failure within 10 years of transplant.^1^ In this paper, we focus on dynamic longitudinal discriminant analysis (LoDA) where patients are classified into one of several prognostic groups (based on future status) using their clinical history, and the classification is updated each time new data becomes available.

Longitudinal discriminant analysis has been developed by a number of authors over relatively recent history. Initial interest focused on using a single continuous longitudinal marker to predict group membership for a patient.^2^, ^6^ Multivariate LoDA with continuous markers have also been considered.^7^, ^9^ However, some of the longitudinal markers may not be continuous, but rather binary or counts. Fieuws et al^1^ presented a multivariate LoDA method for both continuous and binary longitudinal markers. An alternative multivariate LoDA method for longitudinal markers of different types (continuous, counts, and binary), which is robustified against possible model misspecification, was recently developed by Hughes et al.^10^ These models use the longitudinal history of patients of known prognosis to develop a classification procedure that can be used to classify new patients based on their own longitudinal data. For each patient, a probability of belonging to each prognostic group is calculated and used in an allocation scheme to assign the new patient to a group. These probabilities are re‐evaluated for a patient each time new clinical data is available, hence we refer to dynamic LoDA.

In this paper, we aim to further improve the approach of Hughes et al^10^ who applied their method to data from the Standard and New Antiepileptic Drugs (SANAD) study (Marson et al^11^, ^12^), a randomised control trial comparing treatments for patients with newly diagnosed epilepsy. Patients were recruited between December 1999 and August 2004 and underwent regular surveillance. At recruitment, baseline information was collected including the type of epilepsy a patient had, their age, and sex. At regular follow up visits, information was collected regarding the treatment they received, how many seizures the patient had experienced since their previous visit, and what adverse events had been observed. Patients were followed up until potentially January 2006.

The approach of Hughes et al^10^ aimed to identify those patients who will fail to achieve remission from seizures within a 5‐year follow‐up period. Patients who achieve a continuous 12‐month period free from seizures within 5 years of diagnosis are regarded as being in “remission,” whereas patients who do not are referred to as “refractory.” Clinical interest is in being able to assess, each time a patient attends follow‐up, their risk of ultimately belonging to the refractory group. In this approach, a patient's group membership (remission/refractory) does not change and is based on their observed condition at 5 years from initial diagnosis. Good levels of classification accuracy were achieved by Hughes et al,^10^ with sensitivity and specificity showing values above 90%. However, a positive predictive value (PPV) of 59% was reported, which implies that 41% of patients who were classified using the LoDA approach as not achieving remission of seizures did in fact achieve remission. This low PPV was influenced by the relatively low incidence value (only 10% of all the patients were refractory). The clinical implications of wrongly classifying someone as refractory are important (eg, surgery could be considered for these patients). It is hence desirable that a prognostic test performs well, not only in terms of sensitivity and specificity but also in terms of the PPV. In this paper, we propose different allocation schemes that account for patient specific variability and improve the PPV whilst maintaining good levels of specificity and sensitivity.

To formalise our setting, we consider a situation in which regular measurements are made over time (ie, longitudinally) of R⩾1 markers. For each patient, one of *G* prespecified diagnoses is made at a specific future time *T*. This will be represented by a value of the random variable U∈{0,⋯,G−1}, observable only at time *T*. In this way, the population of patients is split into *G* groups depending on their future status at time *T*. In the SANAD example, *G*=2, with *U*=0 denoting the remission group and *U*=1 denoting the refractory group, with *T* being defined as 5 years from initial diagnosis. We denote all the longitudinal observations of a particular marker for a particular patient by $Y_{r}=\left(Y_{r,1},\text{⋯},Y_{r,n_{r}}\right),r=1,\text{⋯},R$ where the observations have been (are to be) recorded at times $t_{r}=\left(t_{r,1},\text{⋯},t_{r,n_{r}}\right)$, $t_{r,1}<\text{⋯}<t_{r,n_{r}}<T$, possibly with additional covariate vectors $v_{r,1},\text{⋯},v_{r,n_{r}}\in R^{p_{r}}$ overall denoted as C. Our aim is to use the information collected about relevant markers to predict the future group, *U*, to which a patient belongs. We aim to do this dynamically, by which we mean that we update our prediction of a patient's future prognosis each time we have new available marker and covariate data for that patient. In other words, for a given *t*, 0<*t*<*T*, let $Y_{r}(t)=\left(Y_{r,j}:\;t_{r,j}⩽t\right)$, *r*=1,…,*R*, $Y(t)=\left(Y_{1}(t),\text{⋯},Y_{R}(t)\right)$ denote the values of the longitudinal markers gathered by time *t*. Similarly, let C(t) denote the covariate information by time *t*. Each time a patient visits a clinic for follow‐up (at time *t*), marker and covariate information is collected leading to Y(t) and C(t), and the prediction Û(t) of the patient's future prognosis is calculated based on available data Y(t) and C(t); that is, the prediction $\hat{U}(t)$ exploits both the newly collected data at time *t* as well as all previously gathered data for that patient.

To calculate the predictions $\hat{U}(t)$, *t*<*T*, we assume that historical data (denoted as Y) are available for patients whose longitudinal marker and covariate history and also their prognosis (group membership) are known. This is used along with the marker and covariate history Y(t) and C(t) of a particular (new) patient to calculate estimates ${\hat{P}}_{g}(t)$, *g*=0,…,*G*−1 of the group membership probabilities $P_{g}(t)=P\left(U=g\;|\;Y(t),C(t),Y\right)$ for this patient. The estimated group membership probabilities ${\hat{P}}_{0}(t),\text{⋯},{\hat{P}}_{G-1}(t)$ are then used to determine the prediction $\hat{U}(t)$ by applying a suitable allocation scheme. Classically, $\hat{U}(t)={\text{argmax}}_{g=0,\text{⋯},G-1}{\hat{P}}_{g}(t)$, but other schemes are possible as well (see section 2.4).

In all of the LoDA procedures referenced above,^1^, ^10^ point estimates ${\hat{P}}_{g}(t)$, *g*=0,…,*G*−1, of the group membership probabilities are used to derive the prediction $\hat{U}(t)$, ie, to assign a new patient to a group. However, the precision of these estimated probabilities may be different for different patients and also at different occasions. This is due to the statistical error of the estimation procedure, which relates mainly to different amount of information borne by the longitudinal marker and covariate information Y(t) and C(t) for different patients and different time points *t*. Indeed, for each individual patient, the amount of useful information to predict the group membership increases with *t* whilst the statistical error of the estimated group membership probabilities decreases. This statistical error can be assessed by using credible/confidence intervals (depending on whether a frequentist or Bayesian methodology is used to fit the underlying models) around the estimated group membership probabilities. We aim to incorporate this additional information in a classification scheme and, thus, reduce the number of false positives and false negatives obtained. The idea is to leave a small number of patients unclassified for whom we are least certain of their prognosis, whilst classifying patients for whom we are confident of their prognosis. We propose a new allocation scheme that takes into account the variability of the estimation of the group membership probabilities using the multivariate LoDA method of Hughes et al^10^ by incorporating information provided by the corresponding credible intervals.

To the best of our knowledge, this is the first time credible (or confidence) intervals for the group membership probabilities have been used in dynamic LoDA, although we are not the first to consider credible intervals in a classification scheme. Komárek and Komárková^13^ do so in the context of longitudinal cluster analysis (where the classification is not “dynamic” and clustering [ie, unsupervised classification] is performed only once using all available longitudinal data) whilst Gugliemli et al^14^ do so in the context of survival analysis (although we note that this is not in a longitudinal context, and there is no sequential updating). Leaving a group as unclassified is similar to the neutral zone classifiers proposed by Zhang et al,^15^ although their unclassified group is based upon an analysis of the misclassification costs. Horrocks and van Den Heuvel^16^ use point estimates to allocate patients to clinical groups and then use credible intervals around the point estimates to help inform patient decisions. Shah et al^17^ calculate a confidence interval around each group membership probability to assess the individual discriminative ability of each patient.

An outline of the remainder of the paper is as follows. In Section 2, we give a brief outline of a multivariate LoDA procedure recently developed by Hughes et al^10^ and provide a review of the most commonly used classification rules. We introduce our new classification scheme based on credible intervals in Section 3. This proposed scheme is applied to the SANAD data in Section 4 where we demonstrate its potential benefits. Section 5 compares our new scheme with the existing classification schemes described in Section 2. Finally, we give a brief discussion of our findings in Section 6.

## 2 LoDA BASED ON A MULTIVARIATE GENERALISED LINEAR MIXED MODEL

### 2.1 Group specific multivariate generalised linear mixed model

Our proposal to classify a new patient on the basis of credible intervals for the individual group probabilities (explained in Section 3) starts from considering the following procedure developed by Hughes *et al.* 10 based on the multivariate generalised linear mixed model (MGLMM) proposed by Komárek and Komárková.^13^ It is assumed that given *U*=*g* (given the allocation of a particular patient into group *g*, *g*=0,…,*G*−1), the values of the longitudinal markers **Y**_1_,…,**Y**_*R*_ are generated by the group specific MGLMM. Namely, given *U*=*g* and given a latent random effects vector $b=\left(b_{1},\text{⋯},b_{R}\right)$, the *j*th longitudinal observation *Y*_*r*,*j*_(*j*=1,…,*n*_*r*_) of the *r*th marker (*r*=1,…,*R*) is assumed to follow a distribution from an exponential family with a dispersion parameter $\phi_{r}^{g}$ and the expectation given as

(1) $$h_{r}^{-1}\left\{E\left(Y_{r,j}\;|\;b,U=g\right)\right\}=x_{r,j}^{g⊤}\alpha_{r}^{g}+z_{r,j}^{g⊤}b_{r},r=1,\text{⋯},R,j=1,\text{⋯},n_{r},$$

where $h_{r}^{-1}$ is a chosen link function; $x_{r,j}^{g}=x_{r,j}^{g}(C)$ and $z_{r,j}^{g}=z_{r,j}^{g}(C)$ are covariate vectors used in a model for the prognostic group *g* derived from the marker information and the available covariates C known by the time at which *Y*_*r*,*j*_ was measured. Parameter vectors $\alpha_{r}^{g}$, *r*=1,…,*R*, *g*=0,…,*G*−1 are unknown regression coefficients.

The random effects vectors **b**_1_,…,**b**_*R*_ are included in the model formula 1 to account for possible correlation between measurements of the same marker on a particular patient. To account also for the correlation between measurements of different markers on a particular patient, a joint distribution with possibly nondiagonal covariance matrix is considered for the overall random effects vector **b**. Parameters of this joint distribution are again group specific. As a certain form of robustification of the model towards misspecification of this distribution, Komárek and Komárková^13^ use a multivariate normal mixture here; that is, it is assumed that

(2) $$b\;|\;U=g∼\overset{K^{g}}{\underset{k=1}{\sum }}w_{k}^{g}\;MVN\left(\mu_{k}^{g},D_{k}^{g}\right),$$

where MVN(μ,D) stands for a multivariate normal distribution with the mean vector ***μ*** and a covariance matrix D having a density denoted as φ(·;μ,D). Unknown parameters of the mixture model 2 in the prognostic group *g* are the mixture weights $w^{g}=\left(w_{1}^{g},\text{⋯},w_{K^{g}}^{g}\right)$( $0<w_{k}^{g}<1$, *k*=1,…,*K* ^*g*^, $\sum_{k=1}^{K^{g}}w_{k}^{g}=1$), the mixture means $\mu_{1}^{g},\text{⋯},\mu_{K^{g}}^{g}$, and the mixture covariance matrices $D_{1}^{g},\text{⋯},D_{K^{g}}^{g}$. The number of mixture components, *K*_*g*_, is assumed to be known.

The MGLMM for group *g*, *g*=0,…,*G*−1, defined by (1) and (2) involves conceptually 2 sets of unknown parameters: $\psi^{g}:=\left(\alpha_{1}^{g},\text{⋯},\alpha_{R}^{g},\phi_{1}^{g},\text{⋯},\phi_{R}^{g}\right),$ which are the regression coefficients and dispersion parameters related to the distribution of the longitudinal response given the random effects and $\theta^{g}:=\left(w^{g},\mu_{1}^{g},\text{⋯},\mu_{K^{g}}^{g},D_{1}^{g},\text{⋯},D_{K^{g}}^{g}\right)$ related to the distribution of random effects. The model induces the following group specific density of the observable vector $\left(Y_{1},\text{⋯},Y_{R}\right)$ of the longitudinal markers

(3) $$f_{g}^{marg}\left(y_{1},\text{⋯},y_{R};\;\psi^{g},\theta^{g},C\right)=\int \overset{R}{\underset{r=1}{\prod }}\;\overset{n_{r}}{\underset{j=1}{\prod }}\;p_{r}\left(y_{r,j}\;|\;b;\;\psi^{g},C\right)\;\overset{K^{g}}{\underset{k=1}{\sum }}w_{k}^{g}\;\varphi (b;\;\mu_{k}^{g},D_{k}^{g})\;db,$$

where $p_{r}\left(\cdot \;|\;b;\;\psi^{g},C\right)$ denotes an exponential family density of the random variable *Y*_*r*,*j*_ related to the GLMM 1.

Several studies, which use discrimination based on mixed models,^9^, ^10^, ^18^ describe classification derived from the group probabilities which use (3) as “marginal” prediction (of a future status of a new patient). Alternatively, in this context, so called “conditional” and “random effects” predictions are discussed, each motivated by considering a different focus on the density of the observed markers. In this paper, we solely focus on the marginal prediction approach, which provided the best predictive accuracy for the SANAD application in Hughes et al.^10^ Nevertheless, the whole methodology described in this paper can be straightforwardly applied for the conditional and random effects prediction.

### 2.2 Individual group probabilities

Suppose first that all model parameters $\psi =\left(\psi^{0},\text{⋯},\psi^{G-1}\right)$, $\theta =\left(\theta^{0},\text{⋯},\theta^{G-1}\right)$ are known and the task is to classify a (new) patient whose history of the longitudinal markers by time *t*<*T* is $Y(t)=\left(Y_{1}(t),\text{⋯},Y_{R}(t)\right)$, $Y_{r}(t)=y_{r}=\left(y_{r,1},\text{⋯},y_{r,n_{r}}\right)$, *r*=1,…,*R* and the covariate information known by time *t* is C(t). By Bayes theorem, we can calculate the following conditional probabilities that the new patient belongs to each of the *G* prognostic groups:

(4) $$\begin{array}{ccc} P\left(U=g\;|\;Y(t),C(t);\;\psi ,\theta \right) & =\frac{\pi_{g}\;f_{g}^{marg}\left(y_{1},\text{⋯},y_{R};\;\psi^{g},\theta^{g},C(t)\right)}{\sum_{\tilde{g}=0}^{G-1}\pi_{\tilde{g}}\;f_{\tilde{g}}^{marg}\left(y_{1},\text{⋯},y_{R};\;\psi^{\tilde{g}},\theta^{\tilde{g}},C(t)\right)} & \\ & =:P_{g}\left(t;\;\psi ,\theta \right),\;\;g=0,\text{⋯},G-1, \end{array}$$

where $\pi_{g}=P(U=g)$, *g*=0,…,*G*−1 are prevalences of the prognostic groups in the study population, which are assumed to be known as is common in applications of discriminant analysis.

In a more common frequentist setting, historical data Y containing both history of the longitudinal markers, covariate information and also information on the group membership of the subjects involved are used to obtain estimates $\hat{\psi }$ and $\hat{\theta }$ of the model parameters, which are then used to calculate the group probabilities of new patients; that is, classification is based on the group probabilities $P_{g}\left(t;\;\hat{\psi },\hat{\theta }\right)$, *g*=0,…,*G*−1.

### 2.3 Bayesian estimates of the individual group probabilities

Hughes et al^10^ suggest to exploit Bayesian estimates of the group probabilities 4 based on the historical data Y for final classification. Those are given as posterior means of (4) with respect to the posterior distribution [ψ,θ|Y] of the model parameters estimated in a Bayesian way using the historical data; that is, allocation of a new patient is based on the following group probabilities:

(5) $$P_{g}(t)=E_{\left[\psi ,\theta \;|\;Y\right]}P_{g}\left(t;\;\psi ,\theta \right)\;g=0,\text{⋯},G-1.$$

Note that the value $P_{g}(t)$ is not only the posterior mean of $P_{g}\left(t;\;\psi ,\theta \right)$ given the historical data Y but can also be expressed as

$$P_{g}(t)=P\left(U=g\;|\;Y(t),C(t),Y\right).\;g=0,\text{⋯},G-1;$$

that is, the group probabilities $P_{0}(t),\text{⋯},P_{G-1}(t)$ express probabilities of allocation of a (new) patient into the *G* prognostic groups given his longitudinal information known by time *t* and also given the information from the historical data. Hughes et al^10^ approximate the group probabilities 5 by a Markov chain Monte Carlo (MCMC) method as

(6) $${\hat{P}}_{g}(t)=\frac{1}{M}\overset{M}{\underset{m=1}{\sum }}\;P_{g}\left(t;\;\psi^{\left(m\right)},\theta^{\left(m\right)}\right),g=0,\text{⋯},G-1,$$

where $\left(\psi^{\left(m\right)},\theta^{\left(m\right)}\right)$, *m*=1,…,*M* is an MCMC sample from the posterior distribution [ψ,θ|Y](see Komárek and Komárková^13^ for details of the MCMC procedure and also for the full specification of the Bayesian model).

### 2.4 Classification rules

The usual procedure is to assign the subject to whichever group has the largest probability; that is, with the estimated group probabilities 6 the prediction $\hat{U}(t)$ of the future status of a (new) patient made at time *t* would be $\hat{U}(t)={\text{argmax}}_{g=0,\text{⋯},G-1}{\hat{P}}_{g}(t)$. As additional observations of the new patient are acquired, the predicted classification of a given patient can be updated by repeating the discriminant analysis step with the new information. The models fitted using the historical data do not need to be recalculated, with the MCMC‐based Bayesian methodology, no additional MCMC samples have to be generated. Simply the estimated group membership probabilities 6 are updated, and the chosen allocation rule is reapplied.

A number of alternative classification rules have been proposed in the statistical literature.^19^ Our focus now will be on the two group classification case (*G*=2), although the methods outlined would work similarly in a multiple group discriminant analysis (*G*>2). For clarity of exposition, we shall refer to the two groups as “disease” (labelled by *U*=1 and also as D) and “disease free” (labelled by *U*=0) groups. The group probabilities 4, 5, and 6, respectively, calculated at time *t* and related to the disease group will be indicated by subscript D.

In the following, it is assumed that prediction of the status of a (new) patient is to be obtained at visit times $0⩽t_{1}<\text{⋯}<t_{n}<T$. We consider the situation where the follow‐up of a particular patient is terminated/changed once it is sufficiently certain that he or she belongs to either of the two groups. Once the clinician is, at time *t*, sufficiently certain that a particular patient belongs to the disease group ( $\hat{U}(t)=1$), future prediction of the disease status of this patient may stop as appropriate clinical procedures are followed to treat the disease. Conversely, it is also common that once the clinician is sufficiently certain that a patient belongs to the nondisease group ( $\hat{U}(t)=0$), there is perhaps no reason to continue in the follow‐up of that patient, making them undertake additional time‐consuming, costly, or uncomfortable medical examinations. However, if neither of the group allocations are certain enough, the patient remains unclassified ( $\hat{U}(t)=\text{N.A.}$) and continues follow‐up till either classification is determined or the disease status is known at time *T*.

As well as the prediction accuracy of the classification procedure, it is also of interest to evaluate the time needed to arrive at a final prediction of the patient's status. This time will be called the “prediction time” *T*_*pred*_ being defined as $T_{pred}=\min\left\{t:\;\hat{U}(t)\neq N.A.\right\}$. The remaining time till the true status of the patient is known will then be called as the “lead time” *T*_*lead*_, ie, *T*_*lead*_=*T*−*T*_*pred*_. If patient remains unclassified after the last examination, we define *T*_*pred*_=*T* and *T*_*lead*_=0 that reflects the assumption that at time *T*, the true disease status is observed.

#### 2.4.1 Dynamic rule

In the two group case, a classical alternative to the simple rule $\hat{U}(t)={\text{argmax}}_{g=0,1}{\hat{P}}_{g}(t)$ that allows us to modify the predictive accuracy measures (such as sensitivity or specificity) is to determine a cutoff value *c* and then to use the classification rule

(7) $$\begin{array}{ccc} & \hat{U}(t)=0,\text{if}\;{\hat{P}}_{D}(t)⩽c, & \\ & \hat{U}(t)=1,\text{if}\;{\hat{P}}_{D}(t)>c. \end{array}$$

Then, indeed, at each visit, classification is determined.

In context of LoDA, this rule was basically considered, eg, by Brant et al^2^ who additionally modified it to reflect a situation where it was more important to detect as early as possible patients in the disease group (as clinical action followed such classification) whereas it was not harmful for patients who truly belonged to the nondisease groups to remain under follow‐up. This leads to the following classification rule. For *j*=1,…,*n*−1:

(8) $$\begin{array}{ccc} & \hat{U}(t_{j})=1,\;\;\;\text{if}\;{\hat{P}}_{D}(t_{j})>c. & \\ & \hat{U}(t_{j})=\text{N.A.},\text{if}\;{\hat{P}}_{D}(t_{j})⩽c. \end{array}$$

For the last visit at *t*=*t*_*n*_, the classical rule 7 is applied and only then patient may be classified as nondiseased. In the following, we will refer to the rule 8 as dynLoDA.

The slightly modified version of the basic rule 7 is

(9) $$\begin{array}{ccc} & \hat{U}(t)=0,\text{if}\;{\hat{P}}_{D}(t)<1-c, & \\ & \hat{U}(t)=1,\text{if}\;{\hat{P}}_{D}(t)>c,\\ & \hat{U}(t)=\text{N.A.},\;\;\text{otherwise}, \end{array}$$

where the cutoff *c*>0.5. Note that only those patients where the group pertinence is likely enough are classified.

#### 2.4.2 Dynamic rule based on 2 consecutive high probabilities

In some situations, getting a single high probability of being in a disease group is not seen as strong evidence to allocate the individual to the disease group, and 2 consecutive high probabilities may be preferable to determine persistent high‐risk patients (see, for example, Reddy et al^20^); that is, under this rule, classification at time *t*_*j*_, *j*=2,…,*n*−1 that directly generalises the rule 8 is given by

(10) $$\begin{array}{ccc} & \hat{U}(t_{j})=1,\;\;\text{if}\;{\hat{P}}_{D}(t_{j})>c\;\&\;{\hat{P}}_{D}(t_{j-1})>c, & \\ & \hat{U}(t_{j})=\text{N.A.},\text{otherwise}. \end{array}$$

At the first visit, the patient's status is always unclassified, ie, $\hat{U}(t_{1})=\text{N.A.}$ irrespective of available data, at the last visit at time *t*=*t*_*n*_, the unclassified case is replaced by classification into the nondisease group.

By using this rule, classification is likely to take longer (leading to larger prediction times) than when using the classification rule based on a single group probability. Nevertheless, it may be necessary in situations where biomarkers used for prediction show large variability.

A further extension of this rule consists of identifying a change in the disease group probability between 2 consecutive visits. For example, if the probability of belonging to the disease group increases by more than a quantity, *k*, then this could be indicative of a worsening condition, and the patient could be classified into the disease group.

#### 2.4.3 Fixed stopping time

Hansen et al^21^ and Lukasiewicz et al^22^ consider scenarios where the aim is to identify nonresponders (diseased patients) at an early date (before outcome is confirmed) whilst maintaining good levels of classification accuracy. They explore a number of fixed visit times and compare classification accuracies obtained by only considering data up until a prespecified time, *T*_*pred*_<*T*; that is, only one prediction is performed at time *T*_*pred*_, and the patient is classified into the disease group if ${\hat{P}}_{D}(T_{pred})>c$; otherwise, the patient is classified as disease free.

#### 2.4.4 Selecting the cutoff

There are a number of ways to choose the cutoff, *c*, of the classification rules outlined above. A typical measure is to use the cutoff associated with the point on the receiver operator characteristic curve nearest to the top left corner. Hence, the cutoff is chosen to minimise *d* ^2^=(1−Sensitivity)^2^+(1−Specificity)^2^. The receiver operator characteristic curve itself, and related quantities are typically calculated using a suitable cross‐validation procedure using the historical data with known group membership. An alternative method would be to use the Youden index.^23^ In this method, the distance between sensitivity plus specificity and the chance line or diagonal line (ie, Sensitivity+Specificity−1) is maximised.

Further options include choosing the cutoff that maximises an alternative measure of classification accuracy such as the probability of correct classification (PCC), PPV, or negative predictive value (NPV). Variations on any of these measures are possible where conditions are made on some of the accuracy measures (eg, Youden index subject to sensitivity of at least 0.8), although such methods require some specification from the investigator. A comparison of 11 different cutoff selection options is given by Freeman and Moisen.^24^

## 3 DYNAMIC CREDIBLE INTERVALS PREDICTION

All classification schemes outlined in section 2.4 are based on point estimates of the disease group probability $P_{D}(t;\;\psi ,\theta )$. In a Bayesian context, the estimated posterior mean 6 is used here. In a frequentist context, one would use $P_{D}(t;\;\hat{\psi },\hat{\theta })$ instead, where $\hat{\psi }$ and $\hat{\theta }$ would be estimates of the model parameters based on the historical data. However, none of the outlined classification rules take into account uncertainty of the model parameter estimators. In a Bayesian context, the group membership probability $P_{D}(t;\;\psi ,\theta )=P\left(U=D\;|\;Y(t),C(t);\;\psi ,\theta \right)$ is just a derived model parameter and its uncertainty is reflected by variability of its posterior distribution. This posterior variability not only accounts for uncertainty attributed to estimation of the model parameters using the historical data but also the amount of information, which is borne by the longitudinal and covariate history Y(t) and C(t) available at time *t* for a (new) patient for whom the prediction is made. See bottom panels of Figures 2 and 3 for illustration of different variability in the posterior distribution of the group membership probabilities for different patients and also over time for one patient.

The variability of the posterior distribution of a quantity estimated in a Bayesian model can be summarised by its (1−*α*)100% credible interval. The (1−*α*)100% credible interval for $P_{D}(t;\;\psi ,\theta )$ is $\left(P_{D}^{LOW}(t),P_{D}^{UPP}(t)\right)$ if

(11) $$P\left[P_{D}(t;\;\psi ,\theta )\in \left(P_{D}^{LOW}(t),P_{D}^{UPP}(t)\right)\;|\;Y\right]=1-\alpha .$$

Condition 11 does not uniquely determine the credible interval, as it only states that the interval captures 1−*α* of the probability mass of the posterior distribution of $P_{D}(t;\;\psi ,\theta )$. If the corresponding posterior distribution is unimodal (as is mostly the case in our setting), then the shortest credible interval is called the highest posterior density (HPD) credible interval, (see section 5.5 in Robert^25^). The HPD interval can easily be calculated once the MCMC sample $\left(\psi^{\left(m\right)},\theta^{\left(m\right)}\right)$, *m*=1,…,*M*, from the posterior distribution [ψ,θ|Y] of the primary model parameters is available. In the following, let $\left(P_{D}^{LOW}(t),P_{D}^{UPP}(t)\right)$ denote the (1−*α*)100% HPD credible interval for $P_{D}(t;\;\psi ,\theta )$.

In summary, the HPD credible interval captures the level of uncertainty of the corresponding group membership probability and provides an indication of the level of confidence one can have in the classification given. We propose a new classification scheme based on the use of credible intervals within the framework of dynamic LoDA. The idea is to incorporate the additional information provided by the credible intervals in a dynamic classification rule to create a procedure that achieves better values of prediction accuracy than procedures based purely on the point estimates.

The newly proposed dynamic credible interval (dynCI) classification rule to predict, at time *t*, the status of a (new) patient, again based on a cutoff value *c*∈(0,1) is as follows:

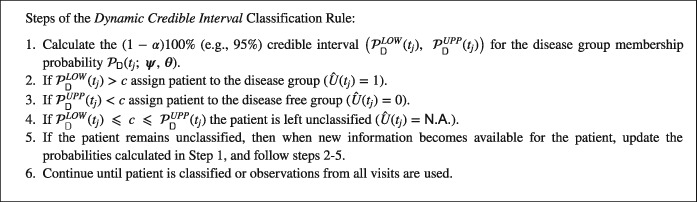

The primary motivation for specification of steps 2 and 3 is to apply the basic rule 7 but only if we are sufficiently certain as expressed by the posterior probability of at least1−*α* that the disease group membership probability is higher then the cutoff *c* (step 2) or lower than the cutoff *c*. The prediction time *T*_*pred*_ for a patient with (scheduled) visits at times $0⩽t_{1}<\text{⋯}<t_{n}<T$ is then

$$T_{pred}=\min\left\{t_{j}:\;P_{D}^{LOW}(t_{j})>c\;\text{or}\;P_{D}^{UPP}(t_{j})<c,j=1,\text{⋯},n\right\}.$$

If the clinical interest is in only one particular group, then classification could focus on the classification conditions of that group, and patients classified in the other group could be “treated” in the same way as the unclassified patients who remain under observation. For example, in the SANAD example presented further in this paper, the allocation scheme has been specifically designed to identify so called refractory patients (as opposed to the remission patients). When a patient is classified as refractory, prediction stops for this patient so that alternative treatment options can be considered. On the other hand, a patient who is classified as being in remission (by step 3 of the rule) remains under observation until they achieve remission or their 5 years (*T*=5 years) since diagnosis is up. So in our case, steps 3 and 4 are combined until the final visit for each patient, and any patient not classified as refractory is treated as unclassified (ie $\hat{U}(t)=\text{N.A.}$). At the final visit considered for each patient (the visit before true status is determined), the patient could be classified as refractory, remission, or, if the status is still unclear, unclassified.

### 3.1 Benefits of using credible intervals

One potential benefit of the dynCI scheme is that some of the false positives and false negatives generated with other schemes may now be left unclassified. In our application, 3 groups of patients are generated under the dynCI scheme, a group of patients confidently classified as refractory, a second group of patients we are confident will achieve remission, and a third group of patients we are unsure how to classify. We may be able to identify some patients about whom we are confident of their classification at an early stage, allowing alternative treatments to be considered much earlier to those patients whilst waiting longer for patients we are less confident about their group membership.

### 3.2 How to treat the unclassified group

An interesting question is what should be done with the unclassified patients. The answer to this depends on what the clinical scenario is. In our SANAD example, the unclassified group and the remission group are equally treated up until the final clinic visit for each patient (when a distinction is made between remission and unclassified patients), as clinicians would simply continue to observe them at regular intervals. One might consider merging the remission group and the unclassified group since the main aim in this case is to be more confident in prediction of patients with refractory epilepsy.

In a screening setting the groups could be used to influence personalised screening schedules. For example, in a study of diabetic retinopathy, the patients predicted as being likely to develop sight threatening diabetic retinopathy could be assigned to more frequent follow up appointments. Those patients we were confident were low risk could be assigned less frequent appointments than currently allocated, whilst the unclassified group could remain on the existing follow up schedule (annual screening intervals).

A different clinical application relates to diagnostic tests. For example, a cheap and non‐invasive diagnostic test that uses data from a number of urine samples taken over time may be offered as an alternative to a more accurate but invasive and expensive diagnostic test (eg, biopsy). In this case, patients identified by the LoDA procedure as belonging to the disease group could be assigned further treatment, whilst patients from the unclassified group would be individuals that the clinicians may wish to offer the invasive test.

## 4 EPILEPSY EXAMPLE

In this section, we explore the merits of the dynCI allocation scheme using data from the SANAD study. In total, 1752 patients were considered of which 1577 were observed to achieve remission within 5 years of diagnosis, whilst 175 were not and hence categorised as refractory. These data have been previously analysed in the context of LoDA by Hughes et al,^10^ and we consider the same model here. We fit a MGLMM consisting of 3 longitudinal biomarkers: a binary marker indicating whether the patient had suffered seizures since their last visit, a continuous marker detailing the number of seizures since the last visit (transformed by *l* *o* *g*(*c* *o* *u* *n* *t*+1)), and a Poisson distributed marker indicating the number of adverse events experienced since the previous clinic visit. The analysis here considers 20 fewer patients than were analysed in Hughes et al^10^ who were later discovered not to have epilepsy and their seizures were related to other causes. Nevertheless, the sensitivities and specificities obtained here are very similar to those reported in Hughes et al,^10^ so we conclude the removal of these 20 patients does not affect the overall accuracy of the LoDA methods.

We use as explanatory covariates the time since the last follow‐up (as the visit schedule is irregular), gender, age at diagnosis of epilepsy, time since diagnosis, type of epilepsy (a binary indicator as to whether the patient has generalised epilepsy or not), and a binary covariate indicating whether or not the diagnosis occurred before the June 6, 2001. This variable was included to reflect the fact that a new drug was added to the trial on this date. To model the random effects, we allow a random intercept and assume that the random effects follow a 2 component normal mixture distribution. More information on the SANAD data used in this analysis can be found in Hughes et al^10^ and Marson et al.^11^, ^12^

We present here the results of a leave‐one‐out cross validation study. For each patient, the MGLMM is fit using all the other patients but excluding the patient for whom prediction is to be made, and then LoDA is used to determine the group membership probabilities at each clinic visit. The cross validation study was set up in this manner to provide a clear picture of the predictions, and their associated variability for each individual patient. Calculations were performed in R,^26^ and the MGLMMs and LoDA were performed using the package mixAK.^27^

### 4.1 Dynamic rule using credible intervals

In this section, we compare the prediction accuracy between the dynLoDA and dynCI classification schemes and investigate the effect that level of credibility has on the accuracy. Specifically, we consider 99%, 95%, 90%, and 50% credible intervals within the dynCI scheme.

Table 1 shows the classification results using the dynLoDA scheme (panel A) and the dynCI scheme for various levels of credibility (panels B‐E). Note that the total number of patients, 1685, is less than the 1752 patients considered. The “missing” 67 patients (3.8%) had only 1 recorded visit, at which their status was confirmed, and therefore, prediction was not possible for these patients. These patients could be used for model fitting however. The optimal cutoff 0.83 is determined by the *d* ^2^ rule of section 2.4. With the exception of the 50% HPD, using the dynCI scheme reduces the number of both false positives and false negatives. This is because a percentage of patients wrongly classified under the dynLoDA scheme is left unclassified when using the dynCI scheme.

**Table 1:** Classification for the marginal prediction with a cutoff of 0.83 using A, the dynLoDA scheme, and the dynCI prediction with a level of B, 99%; C, 95%; D, 90%; and E, 50%

(A)					
		Classification		
		Remission	Refractory	Total
True	Remission	1384	126	1510
Status	Refractory	12	163	175
	Total	1396	289	1685
(B) 99% HPD CI					
		Classification			
		Remission	Refractory	Unclassified	Total
True	Remission	1368	67	75	1510
Status	Refractory	8	151	16	175
	Total	1376	218	91	1685
(C) 95% HPD CI					
True	Remission	1368	90	52	1510
Status	Refractory	9	155	11	175
	Total	1377	245	63	1685
(D) 90% HPD CI					
True	Remission	1368	98	44	1510
Status	Refractory	10	156	9	175
	Total	1378	254	53	1685
(E) 50% HPD CI					
True	Remission	1361	131	18	1510
Status	Refractory	12	162	1	175
	Total	1373	293	19	1685

Abbreviations: CI, credible interval; HPD, highest posterior density.

Table 2 summarises the predictive accuracy of the dynCI schemes with various levels of credibility. At this point, we consider the same cutoff for each scheme to compare directly the effect of using the dynCI scheme. Specificity, sensitivity and PCC of the classified patients increase slightly when using the dynCI scheme. The most noticeable improvement is in the PPV, which increases from 0.56 in the dynLoDA scheme to 0.69 in our 99% dynCI scheme. Lead and prediction times were calculated for patients correctly identified as refractory, and the means are provided in Table 2. Predictions tend to occur later with the dynCI scheme than with the dynLoDA scheme. As can be expected, the higher the level of credibility, the longer it takes to classify a patient. Nevertheless, the cost in delay for using dynCI is less than 4 months in lead time, and there is still a good time gain since accurate predictions are made before the true outcome is clinically confirmed (lead times between 565 and 661 days or between 18 and 22 months).

**Table 2:** A comparison of the prediction accuracy using dynLoDA and dynCI schemes

		dynCI	dynCI	dynCI	dynCI
	dynLoDA	99% HPDCI	95% HPDCI	90% HPDCI	50% HPDCI
Cutoff	0.83	0.83	0.83	0.83	0.83
**Sensitivity (classified data)**	**0.93**	**0.95**	**0.95**	**0.94**	**0.93**
Sensitivity	0.93	0.86	0.89	0.89	0.93
**Specificity (classified data)**	**0.92**	**0.95**	**0.94**	**0.93**	**0.91**
Specificity	0.92	0.91	0.91	0.91	0.90
**PCC (classified data)**	**0.92**	**0.95**	**0.94**	**0.93**	**0.91**
PCC	0.92	0.90	0.90	0.90	0.90
AUC	0.97	0.98	0.98	0.97	0.97
**PPV**	**0.56**	**0.69**	**0.63**	**0.61**	**0.55**
NPV	0.99	0.99	0.99	0.99	0.99
Proportion unclassified	0.00	0.05	0.04	0.03	0.01
Mean lead time (days)	675	565	595	614	661
Mean prediction time (days)	857	972	942	918	872

*Note*. The sensitivities and specificities highlighted in bold are calculated without considering the unclassified patients.

AUC, area under curve; HPDCI, highest posterior density credible interval; NPV, negative predictive value; PCC, probability of correct classification; PPV, positive predictive value.

The overall levels of specificity and PCC decrease slightly using the 99%dynCI scheme and the sensitivity drops from 0.93 to 0.86. Comparison of panels A and B of Table 1 reveals that this drop in sensitivity was due to 16 truly refractory patients remaining unclassified, of whom 12 had been correctly identified using the dynLoDA scheme. Since a higher proportion of correctly identified refractory cases than misclassified refractory cases were moved to the unclassified category the overall sensitivity inevitably drops. However, we would argue that the benefit of the dynCI method is the greater level of confidence obtained for the classified patients (with an increase in PPV of over 10%, from 0.56 to 0.69). In particular, 59 of the false positive cases are now unclassified the clinicians can be more confident in considering further treatment for patients classified as refractory. There is an increase in PPV using the dynCI scheme (except for the 50% HPD), since a higher percentage of patients predicted as refractory are in fact truly refractory, increasing the usefulness of the classification. The gain in PPV, and in other predictive accuracy measures, decreases as the level of credibility decreases. This is to be expected, as the narrowing of the credible interval can be thought of as a convergence towards the mean group membership probability. Hence, we recommend the use of wide credible intervals in our setting.

A small number of patients are in the unclassified group with between 1% and 5% of patients not classified depending on the credibility level (Table 2). As expected, as the level of the credible intervals decreases, the credible intervals narrow, and the number of patients in the unclassified group is reduced. The 99% dynCI scheme increases the specificity from 0.92 (obtained via the dynLoDA rule) to over 0.95 (only those patients who are classified are used in the calculation), whilst the sensitivity increases slightly (from 0.93 to 0.95).

### 4.2 Why use credible intervals?

**Figure 1:**
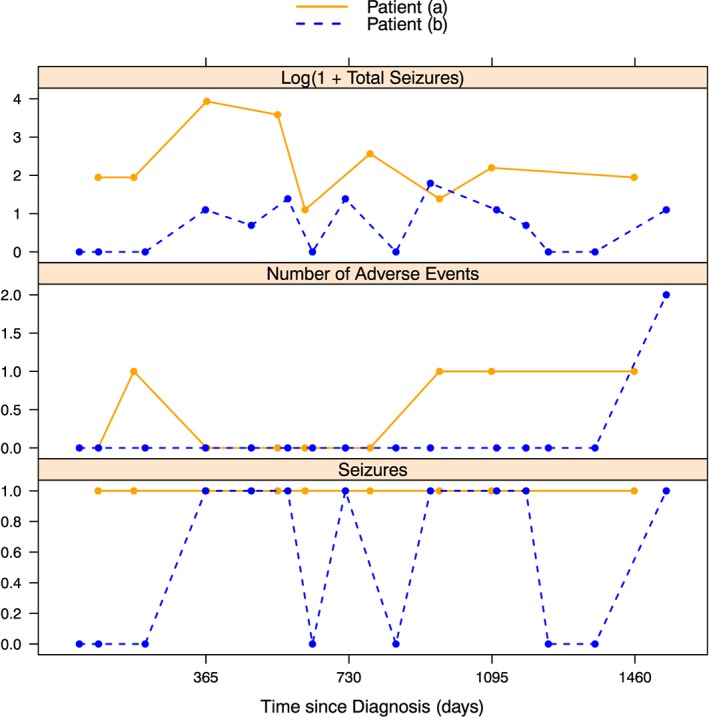
The longitudinal observations of 2 patients from the Standard and New Antiepleptic Drugs Dataset. Patient A was a 17‐year‐old male and patient B was an 8‐year‐old female. Both had generalised epilepsy diagnosed before June 6, 2001 [Colour figure can be viewed at wileyonlinelibrary.com]

**Figure 2:**
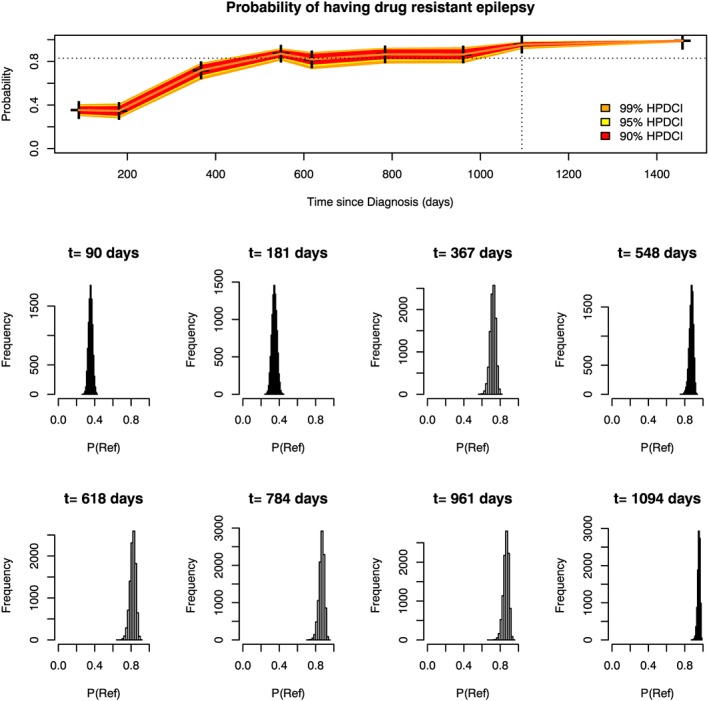
Marginal group membership probabilities over time for patient A (top panel). Histograms showing the posterior distribution of the probability of being in the refractory group are shown for each clinic visit below the top panel. The dotted vertical line denotes the time at which the patient was classified as refractory using the dynCI scheme with 99% credible intervals. The dotted horizontal line shows the required cutoff of 0.83 [Colour figure can be viewed at wileyonlinelibrary.com]

**Figure 3:**
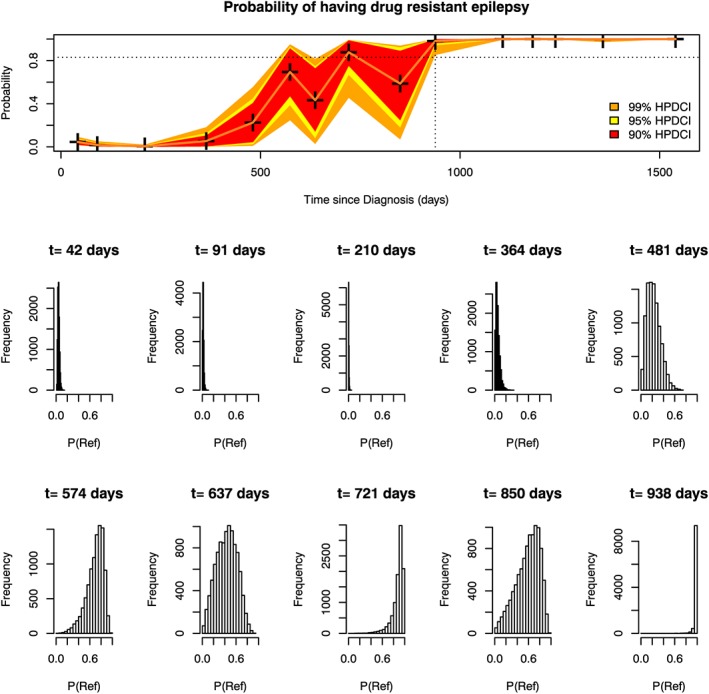
Marginal group membership probabilities over time for Patient B (top panel). Histograms showing the posterior distribution of the probability of being in the refractory group are shown for each clinic visit below the top panel. The dotted vertical line denotes the time at which the patient was classified as refractory using the dynCI scheme with 99% credible intervals. The dotted horizontal line shows the required cutoff of 0.83 [Colour figure can be viewed at wileyonlinelibrary.com]

The schemes in section 2.4 only used, in a Bayesian setting, the posterior mean group membership probabilities to make a decision on a patient's group membership. We suggest that, because of posterior variability of the group membership probabilities, there may be substantial heterogeneity between patients in terms of the credibility of their estimated probabilities (their posterior means). This can be seen by considering 2 refractory patients whose longitudinal data are shown in Figure 1 and probability of being in the refractory group over time is shown for patient A in Figure 2, and for patient B in Figure 3. Histograms reflecting the posterior distribution of the probability of being in the refractory group (obtained from 10 000 realisations of the MCMC sample) are also shown at each clinic visit. The final visit for patient A and the last 5 visits for patient B are not shown as the histograms do not differ much from the last visit shown.

Patient A experienced seizures at each clinic visit. As a result, even very close to the initial diagnosis the probability that he would ultimately be in the refractory group is around 0.4. When the number of seizures experienced increased at the third and fourth visits, his probability of belonging to the refractory group rose significantly to around 0.8. Since he kept having seizures, and his condition did not seem to be improving, it was very likely that he would ultimately be in the refractory group, which is reflected in the fact that the credibility bands are reasonably narrow for patient A and the histograms are all tightly clustered around the mean group membership probability.

Patient B had much more variety in her longitudinal observations. At some visits, she had experienced seizures, whilst at others, she had not. Consequently, her probability of being in the refractory group changed more noticeably over time, in contrast to the steady increase of patient A. Her initial period free of seizures resulted in low probabilities that she would ultimately belong to the refractory group. At her fourth visit, she experienced seizures (but only 2, and no adverse events experienced). However, our model takes into account the full longitudinal history of the patient and not just her current information, so the fact that she had been seizure free until this point resulted in her probability of being refractory being relatively low. Once she had experienced seizures at her subsequent two visits, the probability of being refractory continued to rise, but the uncertainty for this patient, due to her initial good response, was shown in the wide credible intervals and wide histograms (high posterior variability) for visits 5 to 9. Only after she had been observed to have seizures for a longer period does her credible interval begin to narrow (visit 10), allowing her to be correctly identified as refractory.

## 5 COMPARISON OF CLASSIFICATION RULES

Sections 2.4 and 3 describe 4 different allocation schemes, dynCI, dynLoDA, dynLoDA, with 2 consecutive probabilities above a cutoff and a fixed time prediction scheme. Five ways of selecting the cutoff value are discussed, d ^2^, Youden index and maximising PCC, PPV, and NPV. We compare the allocation schemes under each choice of cutoff.

For the fixed time prediction scheme, we consider making a prediction of a patient's status at t_pred_=1,2,3,4 years. At each time point, t_pred_, we focus on patients whose true status has not been determined at that point (we exclude patients who achieved remission before t_pred_). Prediction is made using all the available data for each patient up until t_pred_. For all other allocation schemes, the classification is made dynamically following the rules outlined in section 2.4. A 99% credible interval was used for the dynCI scheme since this gave the best predictive accuracy (Table 2).

Table 3 shows summaries of the classification accuracy using the allocation schemes. Prediction based solely on a fixed time point (1, 2, 3, or 4 years postdiagnosis) yields worse prediction accuracy than when allowing a dynamic prediction scheme. This result indicates the existence of heterogeneity amongst patients. Some patients are easy to identify relatively early, whilst others take longer to determine their status. A dynamic scheme, which predicts patients as refractory at the time we are confident about the prediction, allows flexible stopping rules based on individual patient data.

**Table 3:** Prediction accuracy using different allocation rules

	d ^2^	Youden	Max PCC	Max PPV	Max NPV	d ^2^	Youden	Max PCC	Max PPV	Max NPV
	**dynCI**					**dynLoDA**				
Cutoff	0.83	0.83	0.99	0.99	0.01	0.83	0.83	0.99	0.99	0.01
Sensitivity	0.95	0.95	0.84	0.84	1.00	0.93	0.93	0.77	0.77	1.00
Specificity	0.95	0.95	0.98	0.98	0.11	0.92	0.92	0.97	0.97	0.12
PCC	0.95	0.95	0.97	0.97	0.24	0.92	0.92	0.95	0.95	0.21
AUC	0.98	0.95	0.95	0.95	0.95	0.97	0.97	0.97	0.97	0.97
PPV	0.69	0.69	0.80	0.80	0.16	0.56	0.56	0.77	0.77	0.12
NPV	0.99	0.99	0.98	0.98	1.00	0.99	0.99	0.97	0.97	1.00
Mean mead time, days	565	565	378	378	1427	675	675	432	432	1427
Mean prediction time, days	972	972	1162	1162	108	857	857	1106	1106	107
	**Two Consecutive**					**1 Year**				
Cutoff	0.58	0.49	0.97	0.99	0.01	0.10	0.10	1.00	0.54	0.01
Sensitivity	0.91	0.94	0.65	0.58	1.00	0.83	0.83	0.00	0.16	0.95
Specificity	0.89	0.86	0.98	0.98	0.33	0.73	0.73	1.00	0.96	0.53
PCC	0.89	0.87	0.94	0.94	0.41	0.74	0.74	0.90	0.88	0.57
AUC	0.96	0.96	0.96	0.96	0.96	0.83	0.83	0.83	0.83	0.83
PPV	0.52	0.47	0.79	0.81	0.16	0.26	0.26		0.32	0.19
NPV	0.99	0.99	0.96	0.95	1.00	0.97	0.97	0.90	0.91	0.99
Mean lead time, days	703	799	386	337	1301	1250	1250		1194	1256
Mean prediction time, days	831	735	1148	1202	233	280	280		312	277
	**2 Years**					**3 Years**				
Cutoff	0.31	0.21	0.91	0.95	0.01	0.87	0.77	0.87	0.99	0.16
Sensitivity	0.79	0.87	0.26	0.20	0.98	0.76	0.81	0.76	0.42	0.95
Specificity	0.67	0.62	0.95	0.97	0.29	0.67	0.62	0.67	0.86	0.31
PCC	0.69	0.67	0.80	0.80	0.44	0.71	0.70	0.71	0.67	0.58
AUC	0.80	0.80	0.80	0.80	0.80	0.75	0.75	0.75	0.75	0.75
PPV	0.40	0.38	0.58	0.62	0.28	0.63	0.61	0.63	0.69	0.50
NPV	0.92	0.95	0.82	0.81	0.98	0.79	0.81	0.79	0.67	0.90
Mean lead time, days	929	937	887	866	943	588	593	588	546	615
Mean prediction time, days	599	595	653	662	590	953	947	953	998	927
	**4 Years**									
Cutoff	0.99	0.93	0.91	0.96	0.02					
Sensitivity	0.76	0.89	0.90	0.85	0.99					
Specificity	0.57	0.50	0.49	0.53	0.06					
PCC	0.71	0.77	0.78	0.76	0.71					
AUC	0.70	0.70	0.70	0.70	0.70					
PPV	0.81	0.80	0.80	0.81	0.71					
NPV	0.51	0.67	0.69	0.61	0.80					
Mean lead time, days	248	279	286	266	297					
Mean prediction time, days	1309	1280	1272	1291	1261					

Note. Blank entries represent cases where no patients were classified as refractory. A 99% credibility interval was used for the dynCI scheme.

AUC, area under curve; NPV, negative predictive value; PCC, probability of correct classification; PPV, positive predictive value.

The cost of maximising PPV and NPV is a significant reduction in sensitivity and specificity, respectively. Whether this would be desirable depends on the clinical situation. With the dynLoDA scheme, selecting the cutoff to maximise PCC only yields a 3% increase in PCC compared to the d ^2^ and Youden methods, but the cost is an 18% drop in sensitivity. When using the dynCI scheme, there is a slight increment in sensitivity, specificity, and PCC, as well as a noticeable improvement in the change in PPV (from 0.56 to 0.69 using the d ^2^ rule) compared to the dynLoDA scheme. The cutoff could be chosen to maximise the PPV. However, with the dynCI scheme, this would yield an 11% increase in PPV (0.69‐0.8) but at the cost of an 11% drop in sensitivity and would require an extra 190 days on average (approximately 6 months) to correctly classify refractory patients. The dynCI scheme could be seen as a way of improving PPV without sacrificing good levels of sensitivity, specificity, and PCC. The cost to this, in comparison to the dynLoDA rule, as mentioned in section 4.1, is a delay in time of classification (on average 4 months as the allocation scheme is more conservative).

**Figure 4:**
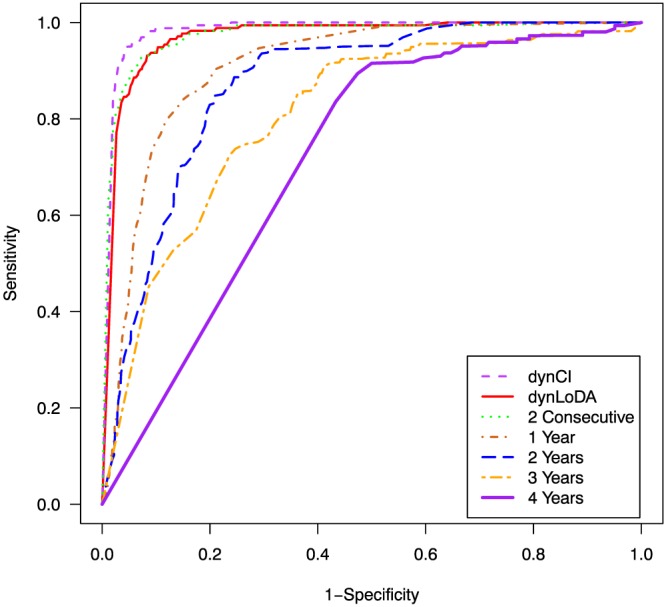
Receiver operating characteristic curves for each classification rule. The dynCI results are based on the classified patients with a 99% credibility interval [Colour figure can be viewed at wileyonlinelibrary.com]

Figure 4 shows the performance of each classification rule over a range of possible cutoff values. The rules based on a fixed prediction time perform substantially worse than those which allow classification to be made dynamically. The dynLoDA and dynLoDA schemes based on 2 consecutive high probabilities perform broadly similarly, although Table 3 shows that using the d ^2^ optimal cutoff, the dynLoDA scheme is slightly better in terms of predictive accuracy. The dynCI scheme with 99% credibility interval shows the best performance.

## 6 DISCUSSION

In this paper, we propose the use of credible intervals for group membership probabilities to improve classification in a dynamic LoDA. The idea is to account for the variability in the level of uncertainty of the group membership probabilities that exists between individuals. The approach proposed here is both dynamic and personalised. It is dynamic since classification is updated each time new data becomes available and personalised because an individual patient's characteristics, both baseline and longitudinal data, but also, individual levels of uncertainty of group membership are taken into account to aid classification. The dynCI model can be fit using the mixAK 27 package in R. We attach as supplementary material a simulated data set and also an R script to fit the MGLMMs and run the LoDA.

We apply our approach to clinical data from the SANAD study to identify in advance patients who will not achieve remission from seizures within 5 years from diagnosis. We show that the use of credible intervals in an allocation scheme can reduce the number of false positives and so increase the PPV of the prognostic rule. Additionally, we have also shown that the sensitivity, specificity, and PCC of classified patients can improve when using credible intervals. Dynamic prediction was considerably more accurate than when prediction was conducted for all patients at a fixed time point.

We demonstrate that the lead time is substantial with the dynCI classification. Therefore, such improvement in lead times could potentially allow clinicians to consider alternative treatment routes for some patients much earlier on than is currently practised, which may improve patients' care. We also show that using credible intervals on the SANAD data allows us to be more confident about the predictions given.

A 99% credible interval was used for the analysis in this paper, as this gave the best improvement in prediction accuracy (Table 2) with only 5% of patients remaining unclassified. The choice of the level of credible interval is application specific and must balance the desire for increased accuracy in prediction with the aim of classifying most patients. How many patients a clinician is willing to leave unclassified depends to some extent on the severity of alternative treatments to be attempted for patients predicted to have the disease. In our epilepsy example, brain surgery might be considered for patients who are classified as refractory. Leaving more patients unclassified would be preferable to wrongly classifying them as refractory and putting them through unnecessary brain surgery. We recommend setting the level of the credible interval as high as possible whilst maintaining an application specific acceptable level of unclassified patients.

In this paper, we have only considered classification into 1 of 2 prognostic groups. The classification scheme based on credible intervals is easily adaptable to a multiple group situation. In that case, a given patient would be classified into group *g* at time *t* if the (estimated) posterior mean ${\hat{P}}_{g}(t)$, Equation 6 is the largest of the posterior means of all the groups and also $P_{g}^{LOW}(t)$, the lower limit of the credible interval for $P_{g}(t;\;\psi ,\theta )$ was greater than a given cutoff. Any patient not meeting this criteria for any of the groups would remain unclassified.

This work considers that once a patient is classified, their status is not revisited. Alternative statistical models may be considered to capture changes in status when individuals may move between the disease and nondisease groups over time (eg, Multistate models, Commenges and Jacqmin‐Gadda^28^ ^, chapter 7^).

We envisage that this work could be used to determine when a patient should next visit a clinic for a screening appointment. Initial work on personalised screening intervals has been done by Rizopoulos et al.^29^ We believe our dynCI rule could be applied so that the unclassified group maintain the currently advised schedule of visits, the disease free group could be allocated less frequent clinic visits, and the disease group could be assigned more frequent clinic visits.

In this paper, we have taken a LoDA approach to classify patient based on their longitudinal data. It would be possible to consider alternative models to classify patients dynamically. However, each approach would require a reframing of the question of interest. For example, one could consider survival‐based approaches such as landmarking^30^ or joint modelling of longitudinal and time to event data.^19^ In this case, the focus would be on risk of achieving remission within the remaining time up until 5 years postdiagnosis. Other alternatives could include machine‐learning methods for high‐dimensional data^31^ and support vector machine learning methods for longitudinal data.^32^ Although this paper considers longitudinal discriminant analysis, we believe that the credible intervals scheme could work well in any dynamic prediction classification scheme including those mentioned above. In principle, any scheme for which credible/confidence intervals are calculated around the probability in question could work well with our scheme.

This work has made used of an HPD credible interval in the allocation scheme. This makes the assumption that the posterior distribution for the group membership probability is unimodal. This can be checked on histograms of sampled values (see Figure 3) that can even be smoothed, eg, by a suitable kernel density estimator (eg, Silverman^33^ ^, chapter 3^) for better insight. It is our empirical experience that unimodality is mostly the case in analysis such as that presented in this paper. However, if the posterior distribution were severely multimodal, then our assumption may lead to inaccurate results since the HPD region could be disjoint intervals (see section 2.3 of Gelman et al^34^). In such a case, it may be preferable to use equal‐tail credible intervals based on posterior quantiles.

Further work could be done to identify more complex stopping rules taking into account severity of alternative treatments, cost of treatment, and possibly other factors. Such models may be more complicated but could allow clinicians to use more information when making decisions for individual patients.

## DATA ACCESSIBILITY

## Supporting information

Supporting info item

Supporting info item

Supporting info item
